# Malignant Phyllodes Tumors of the Breast With Rhabdomyosarcomatous Differentiation: A Case Report and Literature Review

**DOI:** 10.7759/cureus.64361

**Published:** 2024-07-11

**Authors:** Joshua Neposlan, Emily A Goebel, Michael Lock, Robert Dinniwell, Vivian S Tan

**Affiliations:** 1 Department of Radiation Oncology, Schulich School of Medicine & Dentistry, Western University, London, CAN; 2 Department of Pathology and Laboratory Medicine, Western University, London, CAN; 3 Department of Radiation Oncology, Western University, London, CAN; 4 Department of Radiation Oncology, Niagara Health, St. Catharines, CAN

**Keywords:** malignant phyllodes tumor, radiation, case report, breast, rhabdomyosarcomatous

## Abstract

Phyllodes tumor (PT) is a rare fibroepithelial breast neoplasm that is typically graded histopathologically as benign, borderline, and malignant. Malignant PTs (MPTs) exhibit marked stromal cellularity, atypia, overgrowth, increased mitotic activity, and the propensity to metastasize. MPTs represent 10%-15% of all PT cases and often have a notably aggressive disease course. Infrequently, these tumors contain heterologous histological elements, including liposarcoma and fibrosarcoma, among others. Rhabdomyosarcomatous differentiation is an exceptionally rare example of such variation. This report documents the clinical presentation and disease course of a 62-year-old woman diagnosed with MPT with rhabdomyosarcomatous differentiation, just the seventh such confirmed case in the English literature. The patient experienced an arduous disease course, developing metastases to her lungs and axial skeleton just months after her initial diagnosis. Palliative radiation and chemotherapy were initiated, but the patient unfortunately succumbed to her disease just 10 months after the initial diagnosis. This case adds to the scarce literature surrounding the rare development of a heterologous rhabdomyosarcomatous element in an MPT, as well as the decision-making process surrounding the use of radiation to treat such lesions. The details discussed in this paper may inform future approaches for patients diagnosed with this disease.

## Introduction

Phyllodes tumors (PTs) are primary fibroepithelial neoplasms of the breast. Their diagnosis is rare, constituting just 0.3%-1% of all breast tumors and 2.5% of all fibroepithelial breast neoplasms [1]. They are most commonly diagnosed in women, classically in the fourth and fifth decades of life [1].

PTs originate from the breast stroma [2]. Histopathologically, they are identified by their stromal hypercellularity and intracanalicular growth pattern [2]. Characteristically, they can contain leaf-like projections lined with benign ductal epithelium that extend into luminal spaces; however, these are often absent in smaller lesions [3]. Malignant lesions may also contain areas of necrosis or heterologous elements such as rhabdomyosarcoma (RMS) [4]. This study presents a rare case of malignant phyllodes tumors (MPTs) of the breast with rhabdomyosarcomatous differentiation.

## Case presentation

A 62-year-old otherwise healthy female presented to the emergency department in January 2020 via ambulance after a syncopal episode at home. On examination, a large, firm mass was noted in the right breast with overlying skin changes and associated hypervascularity. There was a small opening over the lesion with purulent drainage. The patient endorsed having noticed the mass and associated drainage one week prior and noted that it had grown in size since then. The patient had no personal history of cancer, but family history was significant for breast cancer (aunt) and gastric cancer (father). The patient had a 20-pack-year smoking history.

**Figure 1:**
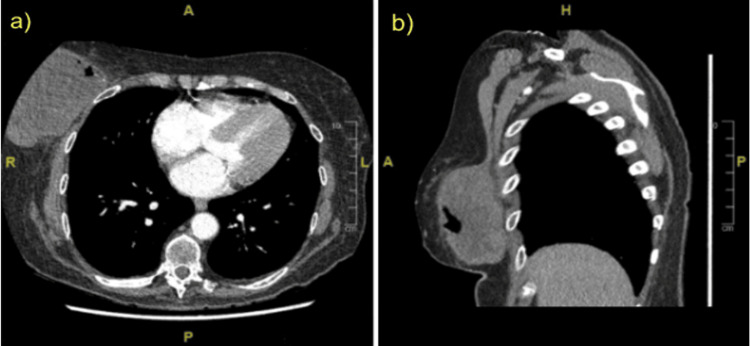
CT chest showing (a) axial and (b) sagittal views of the right breast lesion on initial presentation to the hospital, demonstrating cystic components within the anterior aspect of the mass CT: computed tomography

While initially thought to be mastitis and treated with incision and drainage, an ultrasound of the lesion revealed a complex right breast mass in the 12 o’clock radian. Staging chest/abdomen/pelvis computed tomography (CT) scan revealed a 12-cm mass with clinically negative lymph nodes and no apparent metastases (Figure 1). The contralateral breast exhibited no signs of malignancy on mammography. The needle core biopsy results indicated a likelihood of RMS.

**Figure 2:**
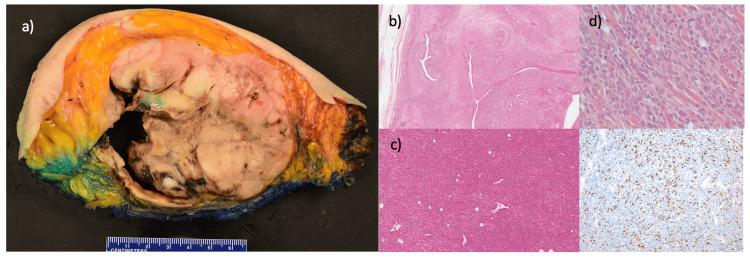
Gross and microscopic features of the patient’s MPTs. (a) Formalin-fixed cross section of the tumor with an ulcerated cavity. (b) Biphasic epithelial and stromal component of the PT (H&E, 100×). (c) Malignant stromal component (H&E, 100×). (d) Malignant stromal component exhibiting cells with striations, which are positive for myogenin immunohistochemistry (inset; H&E, 400×) MPTs: malignant phyllodes tumors; PT: phyllodes tumor; H&E: hematoxylin and eosin

Mastectomy with the removal of the right pectoral muscle revealed a 14.4 cm white, firm, well-circumscribed mass containing a 9.6 cm ulcerated cavity with gray, necrotic tissue (Figure 2a). Microscopically, the tumor was a biphasic fibroepithelial lesion exhibiting stromal overgrowth and predominantly consisted of a highly cellular atypical stroma composed of round to ovoid spindle cells, which contained areas of rhabdomyosarcomatous differentiation characterized by cells with an elongated tail of eosinophilic cytoplasm with striations (Figure 2b). Immunohistochemistry supported rhabdomyosarcomatous differentiation with lesional cells staining positive for desmin and myogenin, and negative for CKAE1/AE3, S100, Melan-A, Sox-10, and CD34. Mitoses were up to 51 per 10 high power fields in the stromal component, and necrosis was present. There were areas of benign ductal epithelium compressed into slit-like spaces by surrounding stroma, distinct leaf-like architecture, and periglandular stromal condensation (Figure 2c). There was no lymphovascular invasion. The tumor invaded the pectoralis muscle and dermis and was completely excised, with the closest posterior margin being 1 mm from the tumor. The final pathology was that of an MPT tumor with extensive rhabdomyosarcomatous differentiation.

**Figure 3:**
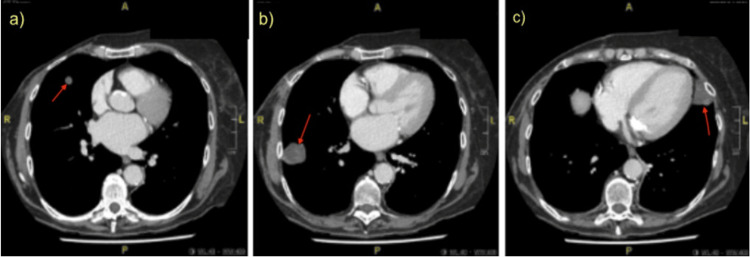
CT chest performed two months after mastectomy. Axial slices show two right lung nodules, measuring (a) 1.2 cm in the upper lobe and (b) 3.3 cm in the lower lobe. (c) A 3.3 cm lesion in the lingular segment of the left lung CT: computed tomography

Following surgery, it was deemed that, given complete surgical excision with negative margins and removal of the pectoral muscle, adjuvant radiation therapy was not indicated, and the patient would have a postoperative follow-up CT scan at six months. However, just over two months after her surgery, the patient presented with new onset pelvic pain, saddle anesthesia, and overflow urinary incontinence. Magnetic resonance imaging of the spine revealed bony metastases in the T10 and L5 vertebral bodies and a 3.2 cm expansile bony lesion in S3. The S3 mass exhibited a mass effect on the S2 nerve root and a moderate-to-severe mass effect on the S3 and nerve roots below it. A bone scan additionally revealed foci in the skull, left clavicle, left 9th rib, sternum, and T1. CT showed bilateral lung metastases (1.2 cm in the right upper lobe, 3.3 cm in the right lower lobe, and a 3.3 cm lesion in the lingular segment of the left lung) (Figure 3). Given the now extensive metastatic disease, the patient began radiation therapy with palliative intent to control her cauda equina-related symptoms. She was administered radiation to a dose of 20 Gy in five fractions to the metastases in her axial skeleton at T10, L5, and S3. This was tolerated well, and she was discharged home, but unfortunately, her disease continued to progress rapidly. A follow-up CT scan two months later revealed interval growth of the T10 vertebral metastasis and three of the pulmonary lesions. Unfortunately, she also experienced further complications in the interim, including pulmonary embolism and hypercalcemia, which were managed over the course of another hospital admission.

Palliative chemotherapy, initially with doxorubicin and later switched to ifosfamide, was initiated, but unfortunately, the disease was refractory to treatment. Progression included involvement of the left deltoid, acromion, clavicle, and sternum, as well as a frontal lobe lesion. The care team and family determined that the risks of chemotherapy at that time outweighed the benefits. The patient was discharged home according to her wishes to live out the rest of the disease course in close proximity to their loved ones. The patient unfortunately succumbed to her disease in November 2020, just 10 months after the initial diagnosis.

## Discussion

Commonly graded along a benign, borderline, and malignant continuum, PTs are differentiated using several histological parameters, including stromal cellularity, stromal atypia, mitoses per 10 HPF, stromal overgrowth, and tumor margin [2]. Most lesions are benign, with just 10%-15% of PTs classified as malignant [1]. Given the lack of widespread consensus regarding specific thresholds within each histologic parameter, there is some discordance in the grading of PTs [5].

Diagnosis of PTs is further complicated by histopathological similarities with a selection of other breast neoplasms. Fibroadenoma, another fibroepithelial breast mass, is the most common differential diagnosis for benign PTs. They can largely be ruled out by the absence of leaf-like stromal projections and hypercellularity of the stroma, with few exceptions [2]. Primary and metastatic sarcomas of the breast are included in the differential diagnosis of MPTs [2]. MPTs with malignant rhabdomyosarcomatous differentiation, as presented in the current case, can be considered the nearest differential to pure RMS of the breast [4]. While generally limited to adolescent females, RMS has also been documented to occur in middle-aged women [6]. Identifying a benign epithelial component within the lesion is highly suggestive of PT and can largely rule out primary breast sarcoma, including RMS [2]. The differential diagnosis of MPT with rhabdomyosarcomatous differentiation further includes metaplastic carcinoma. MPT can have an aggressive clinical course, with a five-year overall survival of 65% [7]. The propensity of PT for local recurrence (LR) or to metastasize correlates with their histopathological grading. It has been suggested that size (over 5 cm) and positive surgical margins also contribute to LR [7]. LR is most common in malignant lesions (23%-30%) but can also occur in benign and borderline cases, with reported incidences of 10%-17% and 14%-25%, respectively [2]. Cases of benign and borderline PTs showing transformation to MPTs on recurrence have been reported [8]. While MPTs have the highest propensity to metastasize (16%), metastases from borderline PTs can occur (<2%) [1]. Metastasis typically occurs through hematogenous spread, while lymph node involvement is extremely rare [9]. The metastatic disease most often affects the lung and skeleton, as was seen in this case, but it can involve all organ systems [2]. Factors that may predispose MPT to metastasis include large size (≥7 cm), positive margins, extensive overgrowth of the stroma, and necrosis [1,7]. The presence of malignant heterologous elements in MPTs confers decreased survival and increased LR [10], but the impact of a specific element on prognosis has not been determined [11].

Management of PTs according to current National Comprehensive Cancer Network guidelines, regardless of grade, is surgical excision via breast-conserving surgery (BCS) with a margin of ≥1 cm, without the need for axillary dissection [12]. However, there is evidence that margins ≥3 mm are associated with a decreased risk of LR in MPTs and that any clear margin is associated with decreased LR in borderline lesions [12]. Benign lesions should also be completely excised; however, positive margins do not confer an increased propensity for LR [12]. BCS does not seem to affect rates of LR in benign lesions compared to mastectomy. Meanwhile, mastectomy has been shown to comparatively reduce LR and increase disease-free survival for lesions classified as borderline or malignant [10]. Furthermore, BCS may not always be feasible or recommended due to factors such as large tumor size and highly malignant features, in which case mastectomy is recommended [13].

There is no standardized framework for the use of adjuvant radiation therapy, but some studies have shown it to reduce LR in borderline and MPT, especially in patients who are young and have tumors less than 5 cm [10,14]. Adjuvant radiation has been used successfully to decrease LR, but not survival, in some patients with borderline or MPTs who have received BCS with margins <1 cm, with cases showing no evidence of LR after 30-72 months of follow-up [10]. This same benefit has not been shown in mastectomy patients [12,14]. There is no evidence of a role for adjuvant chemotherapy in treating PTs of any grade [10,12].

Impact in the context of current literature

This case is just the seventh reported in the English literature to confirm the rhabdomyosarcomatous differentiation of MPTs (Table 1). In this case, the patient presented at a later age than those in previously published studies (62 vs 40-45 years old). Histopathologically, this case shares much in common with those previously reported, exhibiting prominent cross-striations in the rhabdomyoblasts seen on light microscopy and a benign epithelial component compressed into slit-like channels.

**Table 1:** Documented cases of PT with rhabdomyosarcomatous differentiation (histopathological details) F: female; M: male; CXR: chest X-ray; DF: distant failure; NR: not reported; MRI: magnetic resonance imaging; U/S: ultrasound; CT: computed tomography; R: right; L: left; PT: phyllodes tumor

Author	Year	Patient age (sex)	Additional heterologous element(s)	Necrosis (yes/no)	Initial workup reported	Mass details (breast lateralization, dimensions)	Management approach	Clinical outcomes
Barnes and Pietruszka [15]	1978	45(F)	None	Yes	CXR, biopsy	R breast, 9 × 7 × 4 cm	Radical mastectomy	Lung metastases at 24 months (DF). Died at 30 months
Nishimura et al. [16]	1997	80(F)	Osteosarcoma, fibrosarcoma	Yes	Mammogram, U/S, biopsy	R breast, 10 × 9 × 5 cm	Simple mastectomy	Lung and subcutaneous metastases at two months (DF). Died at 10 months
Tan et al. [8]	2005	NR	Liposarcoma	NR	NR	NR	NR	NR
Diwan et al. [17]	2012	40(F)	Osteoclast-like giant cells, spindle cells	Yes	NR	R breast, 15 × 15 × 7 cm	Radical mastectomy	NR
Yadav et al. [4]	2020	45(F)	Osteoclast-like giant cells	Yes	Biopsy	R breast, 14 × 12 × 10 cm	Modified radical mastectomy + adjuvant chemotherapy	No recurrence/metastases at six months
Han et al. [18]	2022	69(F)	None	No	Mammogram, U/S, MRI, biopsy	L breast, 4 × 4 × 3 cm	Radical mastectomy + adjuvant radiotherapy + chemotherapy	No recurrence/metastases at two years
Ali et al. [19]	2023	51(F)	Osteosarcoma	Yes	U/S, biopsy	R breast, 15 × 8 × 7 cm	Total mastectomy	No recurrence/metastases at six months
Our study	2024	62(F)	None	Yes	Mammogram, U/S, CT, biopsy	R breast, 14 × 12 × 8 cm	Radical mastectomy	Lung and spine metastases at two months (DF). Palliative radiation and chemotherapy. Died at 10 months

The axial skeleton and lung involvement described in this case are in keeping with the documented propensity of PTs to metastasize to these regions. However, the rapid progression from diagnosis to the onset of metastatic disease presents a change from those described previously in the literature. Consistent with the cases described by Barnes and Pietruszka [15] and Nishimura et al. [16], the onset of the metastatic disease in our case was associated with rapid progression to death within a matter of months.

## Conclusions

Detailed in this report is a rare case of MPTs with rhabdomyosarcomatous differentiation. The literature surrounding the pathology of and clinical approach to this diagnosis is scarce but growing, a process to which this work contributes. Despite similar histopathology to previously described cases of PT with rhabdomyosarcomatous differentiation in the literature, we document rapid progression to metastatic disease.
